# Supplementary material for: Mapping protein dynamics at high spatial resolution with temperature-jump X-ray crystallography
Source: Nat Chem. 2023 Sep 18;15(11):1549–58. doi: 10.1038/s41557-023-01329-4 (PMC10624634; doi:10.1038/s41557-023-01329-4)
Supplement: Supplementary file 1 — Supplementary Figs. 1 and 2. [file 41557_2023_1329_MOESM1_ESM.pdf]

nature chemistry

Article

https://doi.org/10.1038/s41557-023-01329-4

Mapping protein dynamics at high spatial resolution with temperature-jump X-ray crystallography

In the format provided by the authors and unedited

Supplementary Figures

Supplementary Fig. 1: Pump laser parameters

Supplementary Fig. 2: R-factors from extrapolated structure factor refinement

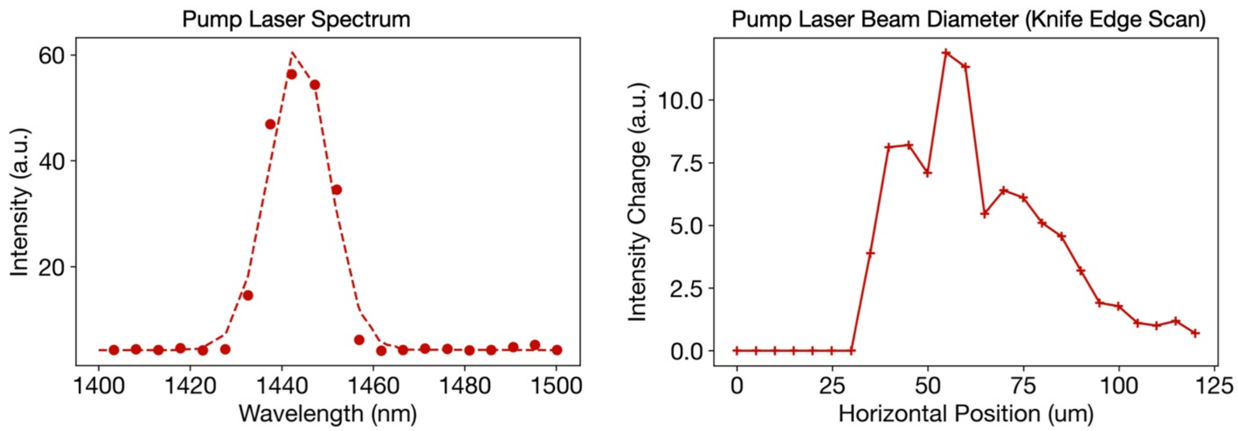

Supplementary Figure 1. Pump laser parameters. Measurements of pump laser spectrum and beam size (horizontal knife edge scan). The spectrum has a peak at approximately 1443 nm with a bandwidth (FWHM) of approximately 16 nm. The results of a knife edge scan reveal the beam to have an approximate diameter (FWHM) of 50 µm. We note these values represent our best estimations, due to the non-gaussian shape of the plotted data. Nevertheless, the IR beam diameter is much larger than that of the X-ray beam, which is approximately 1.5 µm.

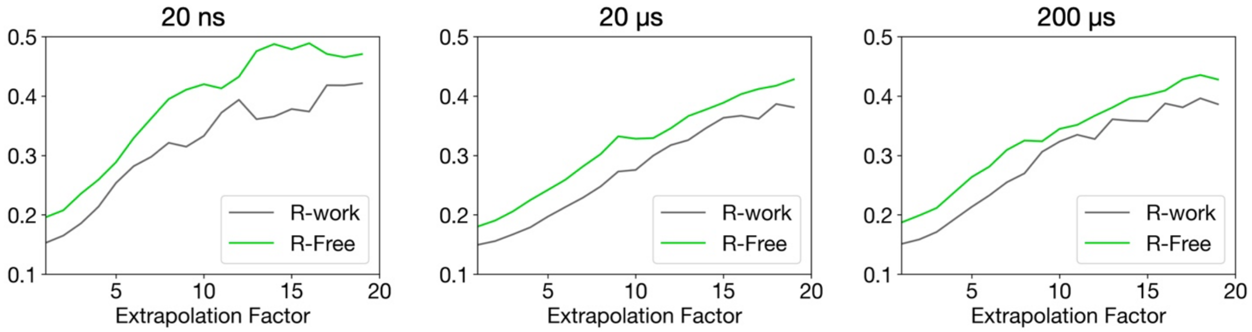

Supplementary Figure 2. R-factors from extrapolated structure factor refinement. Final R-factors following extrapolated structure factor refinement were plotted as a function of extrapolation factor (N as described in Eq. 2) for the three pump-probe time delays for apo lysozyme structures. Both R-work and R-free increased with extrapolation factor for all three datasets.
