# Supplementary material for: Mapping protein dynamics at high spatial resolution with temperature-jump X-ray crystallography
Source: Nat Chem. 2023 Sep 18;15(11):1549–58. doi: 10.1038/s41557-023-01329-4 (PMC10624634; doi:10.1038/s41557-023-01329-4)
Supplement: Supplementary file 2 — Reporting Summary [file 41557_2023_1329_MOESM2_ESM.pdf]

nature portfolio

Corresponding author(s): Michael C. Thompson
Eriko Nango

Last updated by author(s): Jul 21, 2023

Reporting Summary

Nature Portfolio wishes to improve the reproducibility of the work that we publish. This form provides structure for consistency and transparency in reporting. For further information on Nature Portfolio policies, see our Editorial Policies and the Editorial Policy Checklist.

Statistics

For all statistical analyses, confirm that the following items are present in the figure legend, table legend, main text, or Methods section.

n/a	Confirmed	
☐	☒	The exact sample size (n) for each experimental group/condition, given as a discrete number and unit of measurement
☐	☒	A statement on whether measurements were taken from distinct samples or whether the same sample was measured repeatedly
☒	☐	The statistical test(s) used AND whether they are one- or two-sided<br>*Only common tests should be described solely by name; describe more complex techniques in the Methods section.*
☐	☒	A description of all covariates tested
☒	☐	A description of any assumptions or corrections, such as tests of normality and adjustment for multiple comparisons
☐	☒	A full description of the statistical parameters including central tendency (e.g. means) or other basic estimates (e.g. regression coefficient) AND variation (e.g. standard deviation) or associated estimates of uncertainty (e.g. confidence intervals)
☒	☐	For null hypothesis testing, the test statistic (e.g. F, t, r) with confidence intervals, effect sizes, degrees of freedom and P value noted<br>*Give P values as exact values whenever suitable.*
☒	☐	For Bayesian analysis, information on the choice of priors and Markov chain Monte Carlo settings
☒	☐	For hierarchical and complex designs, identification of the appropriate level for tests and full reporting of outcomes
☒	☐	Estimates of effect sizes (e.g. Cohen's d, Pearson's r), indicating how they were calculated

Our web collection on statistics for biologists contains articles on many of the points above.

Software and code

Policy information about availability of computer code

Data collection	Data were collected using custom beamline control software developed at SACLA (Joti, et al. 2015).
Data analysis	Real time data analysis was performed using a pipeline (Nakane, et al. 2016) that calls upon Cheetah (Barty, et al. 2014) for hit finding, and CrystFEL (White, et al. 2012) for indexing and integration. Offline data analysis was performed using cctbx.xfel and cxi.merge for indexing, integration, and merging of the diffraction data (see references, no version numbers available). Structure determination by molecular replacement and refinement was performed using the PHENIX suite (v1.19.2_4158). Additional analysis of electron density and atomic models was performed using custom Python code that is publicly available on our GitHub page (see code availability statement). Code was implemented using Python 3 (v3.8.15), and the following libraries were used: numpy v1.19.2, scipy v1.5.2, pandas v1.1.5, matplotlib v3.3.2, seaborn v0.11.1, gemmi v0.4.5, prody v2.3.1, reciprocalspaceship v0.9.5.

For manuscripts utilizing custom algorithms or software that are central to the research but not yet described in published literature, software must be made available to editors and reviewers. We strongly encourage code deposition in a community repository (e.g. GitHub). See the Nature Portfolio guidelines for submitting code & software for further information.

Data

Policy information about availability of data

All manuscripts must include a data availability statement. This statement should provide the following information, where applicable:

- Accession codes, unique identifiers, or web links for publicly available datasets
- A description of any restrictions on data availability
- For clinical datasets or third party data, please ensure that the statement adheres to our policy

Crystallographic models (refined against the raw structure factors as described above) and the associated reflection data have been deposited in the Protein Data Bank (PDB) under the following accession codes: 8CVU, 8CVV, 8CVW, 8CW0, 8CW1, 8CW3, 8CW5, 8CW6, 8CW7, 8CW8, 8CWB, 8CWC, 8CWD, 8CWE, 8CWF, 8CWG, 8CWH. The model used for MR is also available from the PDB under accession code 1IEE. Additionally, the raw data are publicly available at CXIDB under ID205.

Human research participants

Policy information about studies involving human research participants and Sex and Gender in Research.

Reporting on sex and gender	N/A
Population characteristics	N/A
Recruitment	N/A
Ethics oversight	N/A

Note that full information on the approval of the study protocol must also be provided in the manuscript.

Field-specific reporting

Please select the one below that is the best fit for your research. If you are not sure, read the appropriate sections before making your selection.

☒ Life sciences ☐ Behavioural & social sciences ☐ Ecological, evolutionary & environmental sciences

For a reference copy of the document with all sections, see nature.com/documents/nr-reporting-summary-flat.pdf

Life sciences study design

All studies must disclose on these points even when the disclosure is negative.

Sample size	The number of crystals used to obtain each data set was determined using real time data analysis. Knowing that in serial crystallography, accurate intensity estimation from partial reflections relies on high multiplicity, and that the multiplicity of the merged data tends to decrease as a function of resolution, we continued collecting data until we reached a data multiplicity of >10 for the highest resolution reflections. This threshold was selected, somewhat arbitrarily, based on our team's previous experience processing serial crystallography data. Subsequent data analysis and calculation of CC1/2 and I/sigma(I) quality metrics led to a reduction in the maximum resolution of the data, resulting in an even higher multiplicity for unique reflection measurements from the same number of crystal specimens.
Data exclusions	Diffraction images that could not be fit to the indexed lattice model were discarded.
Replication	Data sets arise from >10,000 individual pump-probe measurements, with controls to remove the effect of covariates (see below). Limited XFEL beamtime precludes the collection of identical data sets for comparison, but the CC1/2 and R-split values reported represent comparisons of random-half datasets.
Randomization	SAmples cannot be randomized (i.e. consecutive measurements necessarily come from the same sample batch), therefore we collected interleaved control measurements to remove the effect of systematic, batch-to-batch variation of samples.
Blinding	Sample blinding is not a standard practice in structural biology.

Reporting for specific materials, systems and methods

We require information from authors about some types of materials, experimental systems and methods used in many studies. Here, indicate whether each material, system or method listed is relevant to your study. If you are not sure if a list item applies to your research, read the appropriate section before selecting a response.

Materials & experimental systems

n/a	Involved in the study
☒	☐ Antibodies
☒	☐ Eukaryotic cell lines
☒	☐ Palaeontology and archaeology
☒	☐ Animals and other organisms
☒	☐ Clinical data
☒	☐ Dual use research of concern

Methods

n/a	Involved in the study
☒	☐ ChIP-seq
☒	☐ Flow cytometry
☒	☐ MRI-based neuroimaging
